# Effect of Dietary Chestnut or Quebracho Tannin Supplementation on Microbial Community and Fatty Acid Profile in the Rumen of Dairy Ewes

**DOI:** 10.1155/2017/4969076

**Published:** 2017-12-31

**Authors:** Arianna Buccioni, Grazia Pallara, Roberta Pastorelli, Letizia Bellini, Alice Cappucci, Federica Mannelli, Sara Minieri, Valentina Roscini, Stefano Rapaccini, Marcello Mele, Luciana Giovannetti, Carlo Viti, Mariano Pauselli

**Affiliations:** ^1^Dipartimento di Scienze delle Produzioni Agroalimentari e dell'Ambiente, University of Florence, Piazzale delle Cascine 18, 50144 Firenze, Italy; ^2^Centro di Ricerca Agricoltura e Ambiente, Consiglio per la Ricerca in Agricoltura e l'Analisi dell'Economia Agraria, Via di Lanciola 12/A, 50125 Firenze, Italy; ^3^Dipartimento di Scienze Agrarie, Alimentari e Agro-Ambientali, University of Pisa, Via del Borghetto 80, 56124 Pisa, Italy; ^4^Dipartimento di Scienze Veterinarie, University of Pisa, Viale delle Piagge 2, 56124 Pisa, Italy; ^5^Dipartimento di Scienze Agrarie Alimentari ed Ambientali, University of Perugia, Borgo XX Giugno 74, 06121 Perugia, Italy

## Abstract

Ruminants derived products have a prominent role in diets and economy worldwide; therefore, the capability to control the rumen microbial ecosystem, for ameliorating their quality, is of fundamental importance in the livestock sector. The aim of this study was to evaluate the effect of dietary supplementation with chestnut and quebracho tannins on microbial community and fatty acid profile, in the rumen fluid of dairy ewes. Multivariate analysis of PCR-DGGE profiles of rumen microbial communities showed a correlation among the presence of chestnut or quebracho in the diet, the specific* Butyrivibrio* group DGGE profiles, the increase in 18:3* cis*9,* cis*12, and* cis*15; 18:2* cis*9 and* cis*12; 18:2* cis*9 and* trans*11; 18:2* trans*11 and* cis*15; and 18:1* trans*11 content, and the decrease in 18:0 concentration. Phylogenetic analysis of DGGE band sequences revealed the presence of bacteria representatives related to the genera* Hungatella*,* Ruminococcus*, and* Eubacterium* and unclassified Lachnospiraceae family members, suggesting that these taxa could be affected by tannins presence in the diets. The results of this study showed that tannins from chestnut and quebracho can reduce the biohydrogenation of unsaturated fatty acids through changes in rumen microbial communities.

## 1. Introduction

The manipulation of rumen microbial ecosystem is considered of primary importance in livestock sciences to improve the feed efficiency and to increase the quality of ruminant-derived products [1]. Recent findings have suggested that tannins, the second most abundant group of plant phenols after lignin, may be used as natural feed additives to modulate rumen fermentation through the inhibition of specific rumen microbial species [2, 3]. Tannins are chemically classified into two groups, hydrolysable and condensed tannins, both able to affect the growth and the metabolic activity of many species of rumen microorganisms [4]. Toxic effects of tannins have been attributed to different mechanisms, such as the inhibition of enzyme activities, the substrate or metal ion deprivation, and the detrimental action on biological membranes [1]. Moreover, tannin effects on rumen microorganisms appear to depend strongly on their chemical structure, their concentration in rumen liquor, and the microbial species involved [5]. For this reason,* in vivo *studies are requested to elucidate the effect of this class of polyphenols on rumen microbial communities and thus their actual employment in ruminant livestock.

Recently, many research studies have been attempted to understand how to increase the concentration of healthful fatty acids (FA) as rumenic acid (RA, 18:2* cis*9,* trans*11) or vaccenic acid (VA, 18:1* trans*11) and transient intermediate of the bacterial biohydrogenation (BH) of polyunsaturated fatty acids (PUFA), in ruminant milk and meat [6–8]. Among ruminal bacteria that appear to be involved in ruminal BH, the* Butyrivibrio* group is particularly sensitive to tannins [2, 3, 9–13]. A selective inhibition of* Butyrivibrio proteoclasticus*, involved in the last step of BH process of linoleic acid (LA, 18:2* cis*9,* cis*12), may provide an accumulation of vaccenic acid (VA, 18:1* trans*11) at the rumen level and, consequently, more RA in ruminant products [11, 14]. Tannins reduced* in vitro* activity and growth of* B. proteoclasticus* [11, 15, 16]. Moreover, literature [2] has shown that the inclusion of quebracho* (Schinopsis lorentzii)* tannins in ewe diet affected the* Butyrivibrio *group in a selective manner and enhanced RA content in dairy products. However, limited information is available on the* in vivo *effect of different sources of tannins on rumen microbiome and FA BH. Therefore, the aim of the present study was to investigate the effect of supplementing ewe diets with chestnut (CHT) or quebracho (QUE) tannin extracts (hydrolysable and condensed tannins, resp.) on rumen liquor FA profile, on the composition of the total rumen bacteria community, and, finally, on the composition of the* Butyrivibrio *group community, by using a polymerase chain reaction-denaturant gradient gel electrophoresis (PCR-DGGE) approach.

## 2. Materials and Methods

### 2.1. Animals and Experimental Design

The experiment was conducted at the Research Centre of the Department of Applied Biology, University of Perugia, Italy. Animals were handled according to the guidelines of the Italian law on animal welfare for experimental animals (Italian Ministry of Health, 2014) and of the University of Perugia Ethics Committee for animal use and care. Three nonlactating Bergamasca x Appenninica ewes (six years old, 60.5 ± 3.4 kg of body weight) equipped with a ruminal cannula of 10 cm internal diameter (Ankom Technology Corp., Macedon, NY, USA) were used. The animals were penned individually. The experiment was conducted as a 3 × 3 Latin square design. Each ewe was fed with the three diets in three consecutive experimental periods of 21 d, including 15 d of adaptation, before each one. At the 21st day the rumen liquor was sampled. The 3 × 3 Latin square was repeated twice with the aim of obtaining more replicates. During the whole experiment, the ewes had free access to water and hay, while the concentrates were administered twice daily (07:30 and 18:30). Orts were collected once daily.

### 2.2. Diets

The experimental diets were the same previously tested in an* in vivo* trial [3]. Diets were composed of chopped grass hay (particle size > 4 cm of length), administered ad libitum and by a concentrate (800 g/head/day), which contained 84.5 g kg^−1^ dry matter (DM) of soybean oil and 52.8 g kg^−1^ DM of bentonite (control, as an inert component to compensate the tannin introduction), or 52.8 g kg^−1^ DM of chestnut tannins (CHT) or 52.8 g kg^−1^ DM of quebracho tannins (QUE). The chemical composition of feeds and the ingredients of concentrates are presented in Table 1. The dose of tannins was chosen to obtain a diet tannin concentration of nearly 1.6% of expected DM intake. On the basis of results from previous studies in literature, this dose was considered as safe for the animal and practical for the farmers [7, 17, 18].

### 2.3. Tannin Sources

Chestnut tannins (750 g kg^−1^ DM of equivalent tannic acid) were provided by Gruppo Mauro Saviola Srl (Radicofani, Siena, Italy), while extract of QUE (456 g kg^−1^ DM of equivalent tannic acid) was provided by Guido Lapi SpA (Castel Franco di Sotto, Pisa, Italy).

Both the extracts were titrated according to Burns [19] to evaluate the equivalent tannic acid. The chemical composition and gas chromatographic profile of CHT were published by Campo et al. [20] and the characteristics of QUE were reported by Vasta et al. [2].

### 2.4. Feed Sampling and Analysis

Samples of feeds were collected daily and stored at −80°C until further analysis. Samples were then ground for chemical analysis by mill Cyclotec 1093 (PBI International, Milan, Italy), using a mesh size of one mm. Concentrations of crude protein (CP), ether extract (EE), and ash were determined according to the AOAC methods 976.06, 920.39, and 942.05, respectively [21]. Neutral detergent fibre (NDF), acid detergent fibre (ADF), and lignin (ADL) contents were determined according to van Soest et al. [22], using heat stable amylase and sodium sulphite, and expressed inclusive of residual ash. Metabolizable energy (ME) and net energy for lactation (NEL) were calculated according to Cannas et al. [23]. Feed FA were extracted according to Folch et al. [24], esterified according to Christie [25] with 19:0 (Sigma Chemical Co., St Louis, MO, USA) as the internal standard, and identified using the same procedure described below for FA of rumen samples.

### 2.5. Rumen Sample Collection and Fatty Acid Profile

Rumen content was sampled from each ewe before morning feeding from two different sites of the rumen and after 21 days of trials on each diet and immediately frozen at −80°C until further analysis.

The FA were extracted according to Folch et al. [24] and methylated according to Buccioni et al. [3]. The FA methyl ester (FAME) composition was carried out by gas-chromatography, according to Buccioni et al. [3]. Individual FAMEs were quantified using valeric acid (5:0) and nonadecanoic acid (19:0) methyl esters (cod W275204 and cod N5377, resp.; Sigma Chemical Co., St. Louis, MO, USA) as internal standards and identified by the comparison of the relative retention times of FAME peaks from samples, with those of the standard mixture 37 Component FAME Mix (C4:0-C24:0, cod 18919-1AMP, Supelco, Bellefonte, PA, USA), individual 18:1* trans*9 and 18:1* trans*11 (cod 46903 and v1381, resp., Sigma-Aldrich, St. Louis, MO, USA), individual 18:2* cis*9,* trans*11 (cod 1255, Matreya Inc. Pleasant GAP, PA, USA), CLA mix standard (cod 05632; Sigma-Aldrich, St. Louis, MO, USA), and published isomeric profile [26–28]. The 18:1 isomers elution sequence was performed according to Kramer et al. [29]. Moreover, standard mix of linolenic acid (LNA) isomers (cod 47792, Supelco, Chemical Co., St. Louis, MO, USA) and of LA isomers (cod 47791, Supelco, Chemical Co., St. Louis, MO, USA) and published isomeric profiles [30] were used to identify the isomers of interest (conjugated *α*-linolenic acid, CALNA, 18:3* cis*9,* trans*11,* cis*15; vaccelenic acid, VLA, 18:2* trans*11,* cis*15). Two bacterial acid methyl ester mixes (cod 47080-U Supelco, Chemical Co., St. Louis, MO; GLC110, Matreya, Pleasant Gap, PA) and individual standard for methyl ester of 14:0* iso*, 14:0* anteiso*, 15:0* iso *and 17:0* anteiso *(cods 21-1211-11, 21-1210-11, 21-1312-11, and 21-1415-11, Larodan Malmo, Sweden) were used to identify branched FA profile. Inter- and intra-assay coefficients of variation were calculated by using a reference standard butter (CRM 164, Community Bureau of Reference, Bruxelles, Belgium) and detection threshold of FA was 0.001 g kg^−1^ of FA [31]. All FA composition results are expressed as g/100 g of FA.

### 2.6. DNA Extraction and PCR Amplification

Total DNA was extracted from 1 mL of each frozen rumen fluid, using the Fast DNA SPIN kit for soil (MP Biomedicals, Santa Ana, CA, USA) with some modifications. Briefly, each sample was thawed and transferred to a 15 mL tube, containing 4.5 mL of a buffer consisting of 150 mM NaCl, 10 mM^−1^ Tris-HCl, pH 8.0, and 10 mM EDTA, vortexed vigorously, and centrifuged at 200*g* at 4°C for 5 min. One mL of supernatant was transferred to a 2 mL centrifuge tube and centrifuged at 14,600*g* at 4°C for 5 min. The pellet was dissolved in 978 *μ*L of buffer sodium phosphate and 122 *μ*L of MT buffer (both solutions are supplied by the Fast DNA SPIN kit for soil, MP Biomedicals, Santa Ana, CA, USA) and then processed, according to the manufacturer's guidelines. DNA yield was quantified by its absorbance at 260 nm and DNA quality was verified using agarose gel electrophoresis (1% w/v).

The extracted DNA was used as a template for PCR amplification of the V6–V8 region of 16S rRNA genes of total bacteria or for the* Butyrivibrio *group. PCR amplifications were conducted using the following primer pairs: F968GC and R1401 for total bacteria (fragment size ~470 bp), according to Nübel et al. [32], and F968GC and B fib for the* Butyrivibrio* group (fragment size ~470 bp), according to Kim et al. [33]. Reactions were carried out using an iCycler Thermal Cycler (Bio-Rad Laboratories, Hertfordshire, UK) in 25 *μ*L volumes containing 1X PCR buffer (67 mM Tris-HCl, pH 8.8, 1.66 mM (NH_4_)_2_SO_4,_ 0.1% Tween-20), 1.5 mM MgCl_2_, 250 *μ*M deoxynucleotide triphosphates (dNTPs), 400 nM of each primer, 1U of Polytaq (Polymed, Florence, Italy), and 10 ng of DNA. PCR reactions were performed under the following conditions: initial denaturation of 94°C for 5 min, followed by 35 cycles of 94°C for 20 s, 56°C for 30 s and 72°C for 45 s, and a final extension of 72°C for 10 min. PCR products were verified by agarose gel (1.2% w/v) ecectrophoresis.

### 2.7. PCR-DGGE Analysis of Total Bacteria and* Butyrivibrio* Group Communities

The PCR amplicons were electrophoretically separated by DGGE on a 6% polyacrylamide gel (acrylamide/bis 37.5:1) in 1X TAE Buffer (40 mM Tris base; 20 mM glacial acetic acid; 1 mM EDTA) using a 50–60% denaturant gradient obtained with a 100% denaturant solution, consisting of 40% v/v deionized formamide, 7 M urea. The gels were run at 60°C and 75 V for 17 h in a Phor-U system (Ingeny International, Goes, NL) and at the end of electrophoretic runs, the gels were stained with SYBR® Gold (Molecular Probes, Eugene, OR) and gel images digitalized using the ChemiDoc XRS apparatus (Bio-Rad Laboratories, Hertfordshire, UK).

### 2.8. Sequence Analysis of PCR-DGGE Fragments

The middle portion of 16 bands selected from* Butyrivibrio *group DGGE profiles was aseptically excised and placed in 20 *μ*L distilled water. The PCR products were eluted through freezing and thawing [34] and reamplified using F968/B fib primer pairs without GC clamp, as previously described. PCR products were checked by DGGE gel electrophoresis and then subjected to directly sequencing by Macrogen Service [35]. The sequence chromatograms were edited using Chromas Lite Software [36] to verify the absence of ambiguous peaks and convert them to FASTA format. The Decipher Find Chimera Web tool [37] was used to uncover chimeras hidden in the 16S rDNA sequences. The BLASTN program [38] available at the NCBI website [39] was used to find taxonomic closely related nucleotide sequences. To increase the accuracy of the assignments, different sequence similarity thresholds were used for different taxonomic levels: a similarity of ≥97% for a species level identification and 95%, 90%, 85%, 80%, and 75% for assignment at the genus, family, order, class, and phylum level, respectively [40].

A phylogenetic dendrogram was constructed to display the apparent relatedness of the partial 16S rRNA gene sequences to each other and to other sequences of equivalent length retrieved from the GenBank database using the software ClustalX 2.0.11 [41] to perform sequence alignment and the software TREECON 1.3b [42] for the construction of the phylogenetic tree using the neighbor-joining method [43]. Bootstrap analysis was performed based on 1000 resamplings.

### 2.9. Statistical Analysis

Statistical analysis was performed using the mixed procedure of SAS [44]. Data were analyzed with the following model:

(1) $$\begin{array}{c} Y_{ijkl}=\mu +A_{i}+P_{j}+D_{k}+\left(\;P_{i}\times D_{k}\;\right)+R_{z}+eijkz, \end{array}$$

where *Y* is the dependent variable, calculated as the mean of the measurements during each sampling period, *μ* is the overall mean, *A*_*i*_ is the random animal effect (*i* = 1 to 3), *P*_*j*_ is the period effect (*j* = 1 to 3), *D*_*k*_ is the diet effect (*k* = 1 to 3), *D*_*k*_ × *P*_*i*_ is their interaction, *R*_*z*_ is the random replicates of the Latin square (*z* = 1 to 2), and *eijkz* is the residual error.

Least squares means estimates are reported. For all statistical analyses, significance was declared at *P* ≤ 0.05.

DGGE profiles were normalized and analyzed using GelCompar II software v 4.6 (Applied Maths, Sint-Martens-Latem, Belgium). The number of bands (species richness) and their relative abundance were used as a proxy of richness and diversity (Shannon index, *H*′, and Simpson index, D) of rumen microbial communities, as described by Pastorelli et al. [45]. The banding profiles of DGGEs, extracted as presence/absence matching tables, were imported into PAST software [46] for multivariate statistical analysis as previously described by Lagomarsino et al. [47]. One-way analysis of similarity (ANOSIM) and permutational multivariate analysis of variance (PERMANOVA) were performed to determine significance differences in the microbial community structure due to the different dietary regimes. In order to find potential connection between community composition and ruminal FA profile and to evidence how these connections may be influenced by the different diets two different canonical correspondence analyses (CCA) were carried out: in the first, the FA assumed to be mainly implicated in BH process (i.e., 18:0, stearic acid, SA; VA; VLA; LA; LNA; RA) were selected; in the second, the FA assumed to be markers of rumen microbial metabolism according to Fievez et al. [48] were considered (i.e., 15:0* iso*; 15:0* ante*; 17:0* iso*; 17:0* ante*). The length and the angle of vectors indicate the relative importance of that FA in discriminating the bacterial community of the different rumen liquors [49]. To identify taxa that mainly contribute to separation of microbial communities, according to the different diets, DGGE band scores were also plotted in the CCA diagrams. The* Butyrivibrio *group DGGE profiles that were mainly related to different FA profile were sequenced.

## 3. Results

### 3.1. Fatty Acids (FA) Composition of Rumen Liquor

The presence of tannins in the diets induced changes in FA profile of rumen liquor. Tannins lowered BH of PUFA leading to an accumulation of linoleic acid (LA), linolenic acid (LNA), and their BH intermediates, reducing the accumulation of stearic acid (SA) (Table 2). In particular, vaccenic acid (VA) and rumenic acid (RA) percentage was significantly higher in rumen liquor from ewes fed with CHT and QUE than in samples from animals fed control diet. Other 18:1 isomers such as* cis*15,* cis*9,* cis*11,* trans*5,* trans*6–8,* trans*9, and* trans*10 showed a similar trend. QUE diet was also associated with an increase of 18:2* trans*10,* cis*12 content in rumen liquor.

Considering the odd and even branched fatty acids, 14:0* iso* content increased only in rumen liquor samples from ewes fed CHT, whereas the content of 15:0* iso* increased in rumen liquor of both tannin-rich diets. However, the content of 15:0* iso* was higher in rumen liquor from ewes fed with QUE. Rumen liquor samples from ewe fed with control and CHT diet had the highest concentration of 17:0* iso *(Table 2). Considering the* ante/iso *FA, the content of 12:0* ante *was significantly higher in CHT samples, whereas 15:0* ante* and 17:0* ante *content was higher in QUE samples.

### 3.2. Effect of Chestnut and Quebracho Tannins on Rumen Microbial Communities

The DGGE banding profiles obtained for total bacteria (Supplementary Material 1) showed a number of bands ranging from 16 to 28. The profiles generated with* Butyrivibrio* group primers were less complex, with a band number of 4–16 (Supplementary Material 2). Richness was not affected by the presence of tannins in the diet in rumen liquor bacterial (*P* = 0.324) and* Butyrivibrio* group (*P* = 0.206) communities. *H*′ index obtained from the DGGE analysis of bacteria (*P* = 0.352) and* Butyrivibrio *group (*P* = 0.117) was similar among treatments and D index did not change significantly in relation to diet in bacterial (*P* = 0.383) or* Butyrivibrio* group communities (*P* = 0.071).

The ANOSIM test applied to 16S rDNA PCR-DGGE profiles showed that the different dietary regimens significantly separated the rumen bacterial communities and that bacterial banding profiles of replicates (6 animal samples × 3 diets) for each diet were more similar to each other (ANOSIM global test *R* = 0.233; *P* < 0.05) than those find when the* Butyrivibrio* group (ANOSIM global test *R* = 0.4216; *P* < 0.01) was analyzed. PERMANOVA analysis confirmed that diet significantly affected the microbial community structure (PERMANOVA global test: bacteria *F* = 2.446, *P* < 0.05;* Butyrivibrio* group *F* = 4.276, *P* < 0.01). PERMANOVA pair-wise test established that bacterial communities under CHT and QUE were significantly different from that of control diet (Table 3) and that for* Butyrivibrio *group communities under CHT were significantly different from the others, whereas the control community was not significantly different to QUE (Table 3).

### 3.3. Bacterial Community Composition in relation to Diet

**Figure 1:**
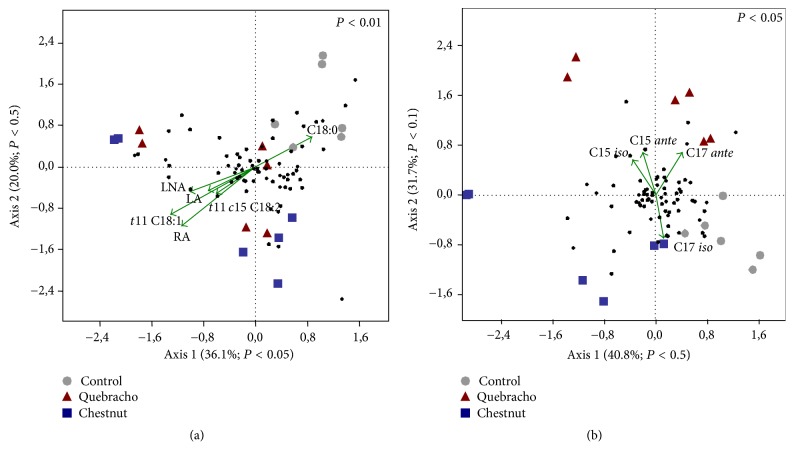
Canonical correspondence analysis (CCA) ordination diagram of ruminal bacterial communities and ruminal fatty acids variables [(a) FA assumed to be mainly implicated in BH process (SA, 18:0; VA, 18:1* trans*11; VLA, 18:1* trans*11,* cis*15; LA; LNA; RA); (b) FA assumed to be markers of rumen microbial metabolism (15:0* iso*; 15:0* ante*; 17:0* iso*; 17:0* ante*)] (vectors) defined by the first and second axes. DGGE band scores were also plotted (black filled circle). For each diagram significance (global test) is reported in upper right side.

**Figure 2:**
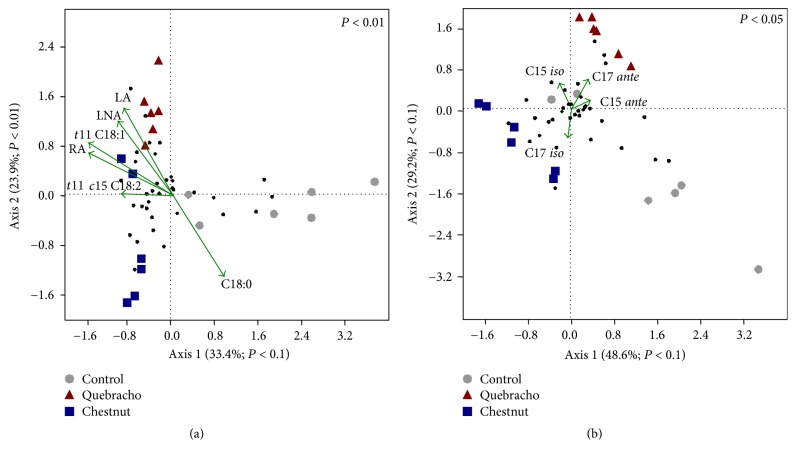
Canonical correspondence analysis (CCA) ordination diagram of ruminal* Butyrivibrio*-related communities (symbols) and ruminal fatty acids variables [(a) FA assumed to be mainly implicated in BH process (SA, 18:0; VA, 18:1* trans*11; VLA, 18:1* trans*11,* cis*15; LA; LNA; RA); (b) FA assumed to be markers of rumen microbial metabolism (15:0* iso*; 15:0* ante*; 17:0* iso*; 17* ante*)] (vectors) defined by the first and second axes. DGGE band scores were also plotted (black filled circle). For each diagram significance (global test) is reported in upper right side.

Canonical correspondence analysis carried out between total bacteria or* Butyrivibrio* group DGGE profiles and the FA assumed to be mainly implicated in BH process (SA; VA, VLA, and LA; LNA; RA) showed that ruminal communities under tannin dietary treatments were separated from the control, Figures 1(a) and 2(a). Similarly, CCA carried out between total bacteria or* Butyrivibrio* group DGGE profiles and the FA assumed to be markers of rumen microbial metabolism (15:0* iso*; 15:0* ante*; 17:0* iso*; 17:0* ante*) according to Fievez et al. [48] indicated that ruminal community under QUE was separated from the control and CHT, Figures 1(b) and 2(b).

Both total bacterial and* Butyrivibrio *group communities under tannin extract diets were positively correlated to LA, LNA, RA, and VA production, Figures 1(a) and 2(a), whereas only those under QUE were positively correlated to C15* ante* and C17* ante*, Figures 1(b) and 2(b). Total bacterial and* Butyrivibrio *group communities of control samples were positively correlated to SA production, Figures 1(a) and 2(a).

### 3.4. Association and Identification of* Butyrivibrio* Group 16S rDNA PCR-DGGE Bands with Key Fatty Acids in the Biohydrogenation Pathway

Multivariate CCA analysis of data generated from* Butyrivibrio *group DGGE allowed identifying bacterial species or groups mainly correlated to a specific FA; thus, bacteria identified by sequencing DGGE bands 1, 2, 3, 4, 5, 6, and 7 (Table 4) were significantly associated with LA, LNA, RA, VA, and VLA (data not shown), whereas bacteria corresponding to bands 8, 9, 10, 11, 12, 13, 14, and 15 (Table 4) were significantly linked with SA (data not shown).

**Figure 3:**
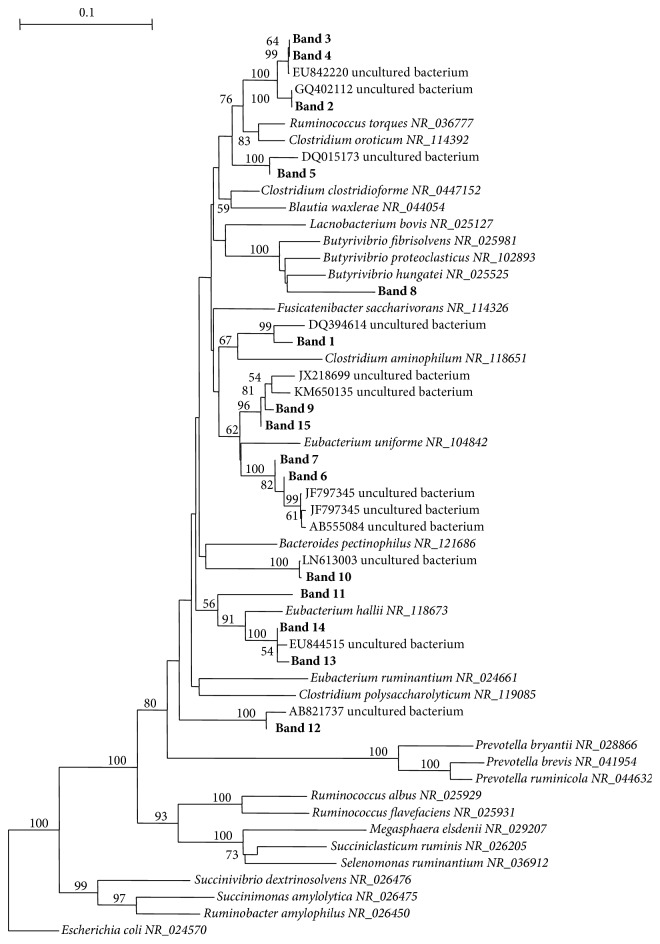
Phylogenetic analysis of* Butyrivibrio* partial 16S rRNA sequences obtained from PCR-DGGE bands using primers F968/B fib and identified species. Sequences obtained in this study are shown in boldface. Bootstrap values of >50% based on 1000 replications are indicated at the nodes. The 16S rRNA gene sequence of* Escherichia coli *(NR_024570) was selected as the outgroup.

Putative taxonomic identification of DNA bands associated with LA, LNA, RA, and VA revealed that they were related to genera* Hungatella *(band 5),* Ruminococcus *(bands 2, 3, and 4), and unclassified Lachnospiraceae (bands 1, 6, and 7; Table 4; Figure 3). Moreover, putative taxonomic identification of bands associated with SA revealed that they were related to unclassified Lachnospiraceae (bands 8, 9, 10, 11, 12, 13, 14, and 15; Table 4; Figure 3).

## 4. Discussion

The BH process of dietary PUFA was strongly lowered by both the tannin-rich diets, regardless of the type of tannin. However, results showed that QUE tannins had a stronger ability to favor the accumulation of BH-intermediate, such as VA and RA, and to reduce the SA concentration in rumen liquor, if compared to CHT tannins. Recently, we found similar results by feeding lactating dairy ewes with diets containing soybean oil supplemented or not with chestnut and quebracho tannins [3]. In the present trial, in rumen liquor* trans*10 isomers of 18:1 and 18:2 also significantly were accumulated in response to tannin supplementation. Previously, in an* in vivo* trial on lactating dairy ewes [3] the administration of the same amount of soybean oil and tannins in the concentrate feed did not result in significant effects on* trans*10 isomers of 18:1 and 18:2, suggesting that in the present trial the rumen environment was more favorable to alternative BH pathway of LA from soybean oil.

The presence of condensed tannins in QUE diet induced the increase of 15:0* ante* and 17:0* ante* content and the decrease of 17:0* iso* content in rumen liquor. Since the first two FA were associated with the growth of cellulolytic strains and the latter with the growth of amylolytic bacteria [48], this pattern or branched FA suggested a detrimental effect of condensed tannins on cellulolytic bacteria. In contrast, the pattern of branched chain FA between rumen liquor samples from CHT and control diets was quite similar, suggesting that CHT tannins did not perturb the growth of cellulolytic bacteria. Indeed, the previous* in vivo* experiment on lactating ewes [3] demonstrated that QUE tannins were more efficient than CHT in limiting cellulolytic bacteria proliferation.

As regards the composition of the whole rumen bacterial community, the diversity indices did not change. As a consequence, changes in the BH pattern could be due to rearrangements of the bacterial species. Indeed, the toxic effect of tannins on specific strains could be compensated by an increase of tannin resistant bacteria in total population. These data are in accordance with previous findings about the selective inhibition of plant extracts containing tannins on the growth and the activity of specific bacterial species representative of rumen microbial populations [2, 11, 50, 51]. Vasta et al. [2], by means of a T-RFLP analysis, demonstrated that dietary supplementation of QUE tannins affected total bacteria community structure in the rumen of lambs fed with QUE supplemented diets. However, it is worth noting that the percentage of tannins employed in the present study was much lower (<2% DM) than that used by Vasta et al. [2]. Thus, putting together data from the present and previous* in vivo* trials on dairy ewes, the addition of practical doses of tannins in the diet is able to cause shifts in the rumen total bacterial community favoring the accumulation of LA and its BH intermediates in rumen liquor.

Bacteria involved in the BH process have been categorized traditionally into two distinct groups: those belonging to group A converting PUFA as LA or LNA into VA and those belonging to group B hydrogenating VA into SA [14]. Group A comprises many known species, among which* Butyrivibrio* spp. are the most important [52, 53], whereas* B. proteoclasticus* is the only known cultivable rumen bacterium belonging to group B [54, 55]. However, recent studies demonstrate that other microorganisms as-yet-uncultivated bacteria phylogenetically classified as* Prevotella*, Lachnospiraceae incertae sedis and unclassified* Bacteroidales*,* Clostridiales,* and Ruminococcaceae might be involved in BH processes with a relevant role [10, 12, 13, 56]. Our data from DGGE analysis showed an effect of 2% DM of QUE or CHT tannins on the composition of the* Butyrivibrio *group.* Butyrivibrio *species are particularly sensitive to condensed tannins [16]. Indeed, these phytochemicals can penetrate the cell wall of* Butyrivibrio *and other Gram positive bacteria and selectively inhibit the cell wall biosynthesis [50]. However, at the same time, remarkable differences among* Butyrivibrio *species were observed in the level of their sensitivity to tannins [11, 50]. The persistence and the appearance of some bands but not of other ones in the* Butyrivibrio *group DGGE gel are consistent with these previous findings.

Multivariate statistics allowed the selection of DGGE bands representative of* Butyrivibrio *group that are putatively involved in the BH process* in vivo*. Seven bands disappeared when ewes were fed with QUE enriched diet, and this effect was associated with reduced rumen SA concentrations. Therefore, it is possible that this group of DNA bands might be representative of other bacteria that play a role in the BH of VA into SA, confirming the findings of several studies [10, 12, 13, 56]. Indeed, the bands here identified are highly related to uncultured rumen bacteria belonging to the family Lachnospiraceae. Interestingly, no sequences were identified as* B. proteoclasticus*, which according to literature is the only known bacterial species able to efficiently biohydrogenate PUFA into SA in the rumen. However,* in vivo* studies have shown contrasting results, since a clear relationship between the reduced amount of* B. proteoclasticus *and a decreased production of SA in rumen liquor was found only in a limited number of trials [2, 3]. A possible explanation to our data may be that the concentration of tannins used in this study was not able to modify* B. proteoclasticus *growth in the rumen but only lowered its capacity to hydrogenate 18:1* trans *FA, as previously suggested by Boeckaert et al. [10]. Otherwise, it is possible that* B. proteoclasticus *has a limited contribution to SA formation* in vivo* and that other yet not known species may have a more important role in this step of the BH pathway. Since only a limited number of rumen species is presently known, this hypothesis is likely. Moreover, it is in agreement with the opinion of other authors who evaluated the effect of marine algae [10] and fish oil [56] on rumen bacterial diversity, evidencing a possible relation between the disappearance of many uncultivated Lachnospiraceae strains, genetically distant from* B. proteoclasticus, *and a significant decrease of rumen SA concentration.

Our study evidenced also that the two types of tannins induced an increase in LA, LNA, RA, and VA and 18:2* trans*11 and* cis*15 in rumen liquor and this was associated with a higher intensity of seven bands in the* Butyrivibrio* group DGGE profiles. Phylogenetic analysis revealed that these sequences were representative of species belonging to genera* Hungatella*,* Ruminococcus*,* Eubacterium* and to unclassified Lachnospiraceae. Once more, these data confirm that other* Butyrivibrio* groups may be involved in the BH pathway and that their increased amount may promote the accumulation of 18:1 intermediates in the rumen. The employment of other and more powerful molecular techniques, such as functional metagenomics, could be useful to clear the role of these uncultured bacteria in the BH pathway.

## 5. Conclusions

The use of chestnut and quebracho tannins in the diet of dairy ewes at a level below 2% DM reduced the extent of ruminal BH process, lowering SA concentration and enhancing the percentage of LA, LNA, VA, RA, and other 18:1 isomers. The changes observed in the FA profile were associated with changes in total bacteria and* Butyrivibrio *group communities, even if they were more evident in presence of quebracho. Bands that disappeared or increased in presence of tannins in the* Butyrivibrio *group DGGE profiles were related to many uncultivated species of Lachnospiraceae, suggesting that these yet not known species may play a role in BH of PUFA. Our study indicates that chestnut and quebracho tannins offer an interesting possibility of modulating favorably rumen bacterial lipid metabolism toward precursors of healthful FA, which are produced in mammary tissues of lactating ewes during milk fat synthesis.

## Figures and Tables

**Table 1:** Ingredients, chemical composition, and fatty acids profile of the experimental concentrates and of the hay and rolled barley administered to the ewes.

	Grass hay	Rolled barley	Control diet	CHT diet	QUE diet
*Ingredients (g kg* ^−1^ * of DM)*					
Barley			213.8	213.8	213.8
Corn			211.3	211.3	211.3
Wheat bran			158.5	158.5	158.5
Soybean meal (44 CP)			126.8	126.8	126.8
Beet pulp			89.8	89.8	89.8
Soybean oil^1^			84.5	84.5	84.5
Bentonite			52.8	-	-
Chestnut tannin extract^2^			-	52.8	-
Quebracho tannin extract^3^			-	-	52.8
Molasses			41.3	41.3	41.3
CaCO_3_			10.6	10.6	10.6
Sodium bicarbonate			5.3	5.3	5.3
Dicalcium phosphate			5.3	5.3	5.3
*Chemical composition (g kg* ^−1^ *of DM)*					
Organic matter	847.0	859.9	816.9	858.1	869.6
Crude protein	111.2	121.0	165.6	173.7	170.3
Ether extract	12.0	16.1	109.4	105.4	102.4
NDF	636.4	134.1	174.7	181.4	172.1
ADF	501.3	54.2	77.6	72.4	74.3
ADL	105.7	14.9	10.6	13.3	8.7
Ash	69.6	21.0	84.6	39.9	39.4
ME (MJ kg^−1^ DM)	7.8	9.9	13.1	14.1	14.1
NEl (Mcal kg^−1^ DM)	0.9	1.2	2.0	2.1	2.1
*Fatty acids (g/100 g of total fatty acids)*					
16:0	35.5	18.2	14.0	14.4	14.9
SA, 18:0	5.8	4.6	3.6	3.4	3.4
18:1 *cis*9	9.3	21.2	23.3	22.9	22.0
LA, 18:2 *cis*9 *cis*12	28.5	45.0	51.4	51.7	51.8
LNA, 18:3 *cis*9 *cis*12 *cis*15	2.8	6.0	5.8	5.6	5.8
Other FA	18.1	4.9	1.9	2.0	2.1

^1^Fatty acid profile of soybean oil (g/100 g of total fatty acids): C16:0, 11.01; C18:0, 3.6; C18:1 *cis*9, 22.09; C18:2 *cis*9 and *cis*12, 53.7; C18:3 *cis*9, *cis*12, and *cis*15, 7.2.  ^2^Hydrolysable tannins extracted from chestnut wood (*Castanea sativa* Mill.) containing 750 g of equivalent tannic acid/kg DM (provided by Gruppo Mauro Saviola Srl Radicofani, Siena, Italy).  ^3^Condensed tannins extracted from quebracho *(Schinopsis lorentzii)* containing 456 g of equivalent tannic acid/kg DM (provided by Guido Lapi SpA, Castel Franco di Sotto, Pisa, Italy).

**Table 2:** Fatty acid profile of rumen liquor from sheep fed with 800 g/head/day of a concentrate containing 84 g kg^−1^ DM of soybean oil plus 0 (control) or 52.8 g kg^−1^ DM of a chestnut tannin extract (CHT) or 52.8 g kg^−1^ of DM of quebracho tannin extract (QUE).

FA g/100 g of total fatty acids	Control	CHT	QUE	SEM^1^	*P* value^2^
10:0	0.197 b	0.186 b	0.439 a	0.077	0.0473
12:0 *ante*	0.237 b	0.387 a	0.261 b	0.041	0.0402
12:0	0.297 b	0.318 b	0.656 a	0.087	0.0213
13:0	0.305 c	0.453 b	0.542 a	0.024	<0.0001
14:0 *iso*	0.317 b	0.613 a	0.404 b	0.068	0.0184
14:0	0.485 b	0.556 b	1.438 a	0.076	<0.0001
15:0 *iso*	0.116 c	0.185 b	0.239 a	0.020	0.0030
15:0 *ante*	0.378 b	0.376 b	0.507 a	0.039	0.0499
15:0	0.776 b	0.605 c	1.054 a	0.017	<0.0001
16:0 *iso*	0.175 b	0.178 b	0.276 a	0.029	0.0353
16:0	10,428 c	17.423 b	23.108 a	0.712	<0.0001
17:0 *iso*	7.544 a	7.418 a	6.135 b	0.288	0.0039
17:0 *ante*	0.189 b	0.154 c	0.247 a	0.010	<0.0001
17:0	0.327 b	0.342 b	0.532 a	0.037	0.0013
SA, 18:0	50.447 a	44.616 b	32.770 c	1.307	<0.0001
18:1 *trans*5	0.051 c	0.313 a	0.132 b	0.055	0.0122
18:1 *tran*6–8	0.476 c	0.682 b	1.134 a	0.131	0.0051
18:1 *trans*9	0.334 c	0.602 b	0.665 a	0.016	<0.0001
18:1 *trans*10	0.681 b	1.254 a	1.359 a	0.147	0.0071
VA, 18:1 *trans*11	1.922 c	6.304 b	7.589 a	0.244	<0.0001
18:1 *trans*12	0.535 c	0.792 b	1.299 a	0.033	<0.0001
18:1 *cis*5	0.374 b	0.697 a	0.799 a	0.069	0.0007
18:1 *cis*7	0.510 c	1.297 b	1.498 a	0.064	<0.0001
18:1 *cis*9	2.337 c	3.879 b	5.340 a	0.111	<0.0001
18:1 *cis*11	0.414 c	0.702 b	0.892 a	0.014	0.0137
18:1 *cis*12	0.258	0.315	0.363	0.042	0.1927
VLA, 18:2 *trans*11, *cis*15	0.123	0.181	0.179	0.049	0.5901
LA, 18:2 *cis*9, *cis*12	0.845 c	1.096 b	1.926 a	0.075	<0.0001
LNA, 18:3 *cis*9, *cis*12, and *cis*15	0.305 c	0.363 b	0.467 a	0.023	0.0004
RA, 18:2 *cis*9, *trans*11	0.651 c	2.137 b	2.600 a	0.042	<0.0001
CLA *trans*10, *cis*12	0.163 b	0.162 b	0.258 a	0.017	0.0008
20:0	0.230 b	0.323 b	0.637 a	0.025	<0.0001
20:4	0.738	1.016	0.959	0.110	0.1855
22:0	0.144 c	0.226 b	0.399 a	0.021	<0.0001

^1^Standard error mean; ^2^probability of significant effect (a, b, and c for *P* < 0.05).

**Table 3:** *P* values from PERMANOVA pair-wise comparison of band profiles from 16S rDNA bacterial DGGE (in boldface, upper right side) and from 16S rDNA *Butyrivibrio* group DGGE (in italics, lower left side).

Diet	Control	CHT	QUE
Control		**0.0450** ^a^	**0.0272** ^a^
CHT	*0.0196* ^a^		**0.1310**
QUE	*0.0737*	*0.0046* ^a^	

^a^Significant value (*P* < 0.05).

**Table 4:** Identification of the selected polymerase chain reaction denaturing gradient gel electrophoresis (16S rDNA PCR-DGGE) fragments.

PCR-DGGE band	Nearest match (GenBank accession number; % sequence similarity)	Relative taxonomic classification
(1)	*Clostridium aminophilum* (NR_118651.1; 93%)	Unclassified Lachnospiraceae
(2)	*Ruminococcus torques *(NR_036777.1; 95%)	*Ruminococcus *spp.
(3)	*Ruminococcus torques (*NR_036777.1; 95%)	*Ruminococcus *spp.
(4)	*Ruminococcus torques (*NR_036777.1; 96%)	*Ruminococcus *spp.
(5)	*Clostridium clostridioforme* (NR_0447152; 95%)	*Hungatella *spp.
(6)	*Fusicatenibacter saccharivorans *(NR_114326.1; 93%)	Unclassified Lachnospiraceae
(7)	*Eubacterium uniforme* (NR_104842.1; 94%)	Unclassified Lachnospiraceae
(8)	*Butyrivibrio proteoclasticus *(NR_102893.1; 94%)	Unclassified Lachnospiraceae
(9)	*Clostridium oroticum *(NR_114392.1; 94%)	Unclassified Lachnospiraceae
(10)	*Blautia waxlerae *(NR_044054.1; 93%)	Unclassified Lachnospiraceae
(11)	*Roseburia intestinalis *(NR_027557.1; 92%)	Unclassified Lachnospiraceae
(12)	*Bacteroides pectinophilus* (NR_121686.1; 90%)	Unclassified Lachnospiraceae
(13)	*Eubacterium hallii* (NR_118673.1; 93%)	Unclassified Lachnospiraceae
(14)	*Eubacterium hallii* (NR_118673.1; 93%)	Unclassified Lachnospiraceae
(15)	*Clostridium oroticum *(NR_114392.1; 94%)	Unclassified Lachnospiraceae

## Abbreviations

ADF: Acid detergent fibre
BH: Biohydrogenation
CLNA: Conjugated linolenic acid
CHT: Chestnut tannins
DM: Dry matter
CP: Crude protein
EE: Ether extract
FA: Fatty acids
FAME: Fatty acid methyl esters
LA: Linoleic acid
LNA: Linolenic acid
ME: Metabolizable energy
NEL: Net energy for lactation
NDF: Neutral detergent fibre
PUFA: Polyunsaturated fatty acids
QUE: Quebracho tannins
RA: Rumenic acid
SA: Stearic acid
VA: Vaccenic acid
VLA: Vaccelenic acid.

## Supplementary Materials

Supplementary Materials Supplementary Material 1: DGGE profiles of 16S rDNA PCR products obtained from DNA extracted from rumen liquor using primer for the total bacteria (F968GC-1401R). CTR, control diet (84 g kg^−1^ DM of soybean oil); CHT, chestnut tannins diet (84 g kg^−1^ DM of soybean oil plus 52.8 g kg^−1^ DM of a chestnut tannin extract); QUE, quebracho tannins diet (84 g kg^−1^ DM of soybean oil plus 52.8 g kg^−1^ DM of a quebracho tannin extract); M, marker used for normalization of bands. Supplementary Material 2: DGGE profiles of 16S rDNA PCR products obtained from DNA extracted from rumen liquor using primer for the* Butyrivibrio* group (F968GC-B fib). CTR, control diet (84 g kg^−1^ DM of soybean oil); CHT, chestnut tannins diet (84 g kg^−1^ DM of soybean oil plus 52.8 g kg^−1^ DM of a chestnut tannin extract); QUE, quebracho tannins diet (84 g kg^−1^ DM of soybean oil plus 52.8 g kg^−1^ DM of a quebracho tannin extract); M, marker used for normalization of bands.
