# Supplementary material for: Effect of Dietary Chestnut or Quebracho Tannin Supplementation on Microbial Community and Fatty Acid Profile in the Rumen of Dairy Ewes
Source: Biomed Res Int. 2017 Dec 31;2017:4969076. doi: 10.1155/2017/4969076 (PMC5804114; doi:10.1155/2017/4969076)
Supplement: Supplementary Materials — Supplementary Material 1: DGGE profiles of 16S rDNA PCR products obtained from DNA extracted from rumen liquor using primer for the total bacteria (F968GC-1401R). CTR, control diet (84 g kg−1 DM of soybean oil); CHT, chestnut tannins diet (84 g kg−1 DM of soybean oil plus 52.8 g kg−1 DM of a chestnut tannin extract); QUE, quebracho tannins diet (84 g kg−1 DM of soybean oil plus 52.8 g kg−1 DM of a quebracho tannin extract); M, marker used for normalization of bands. Supplementary Material 2: DGGE profiles of 16S rDNA PCR products obtained from DNA extracted from rumen liquor using primer for the Butyrivibrio group (F968GC-B fib). CTR, control diet (84 g kg−1 DM of soybean oil); CHT, chestnut tannins diet (84 g kg−1 DM of soybean oil plus 52.8 g kg−1 DM of a chestnut tannin extract); QUE, quebracho tannins diet (84 g kg−1 DM of soybean oil plus 52.8 g kg−1 DM of a quebracho tannin extract); M, marker used for normalization of bands. [file 4969076.f1.pdf]

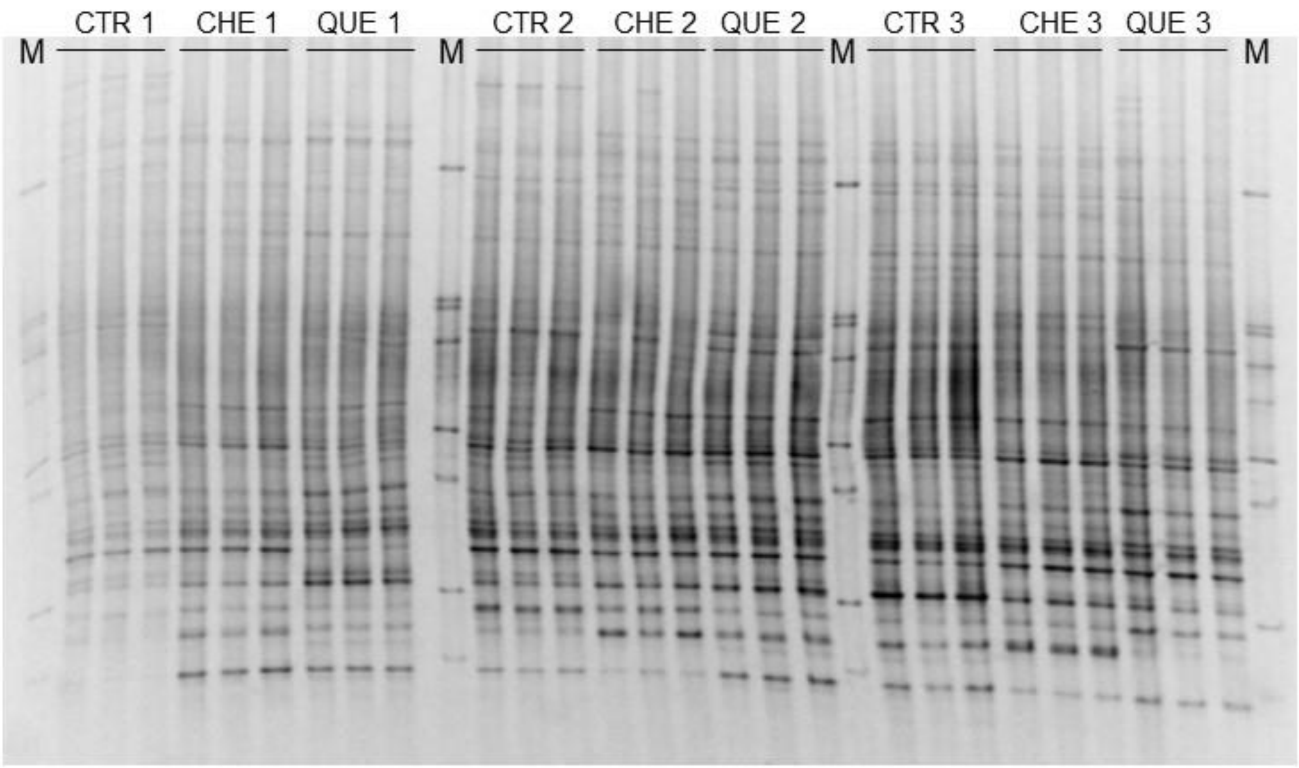

Supplementary material 1. DGGE profiles of 16S rDNA PCR products obtained from DNA extracted from rumen liquor using primer for the total bacteria (F968GC-1401R). CTR, control diet (84 g kg^{-1} DM of soybean oil); CHT, chestnut tannins diet (84 g kg^{-1} DM of soybean oil plus 52.8 g kg^{-1} DM of a chestnut tannin extract); QUE, quebracho tannins diet (84 g kg^{-1} DM of soybean oil plus 52.8 g kg^{-1} DM of a quebracho tannin extract); M, marker used for normalization of bands.

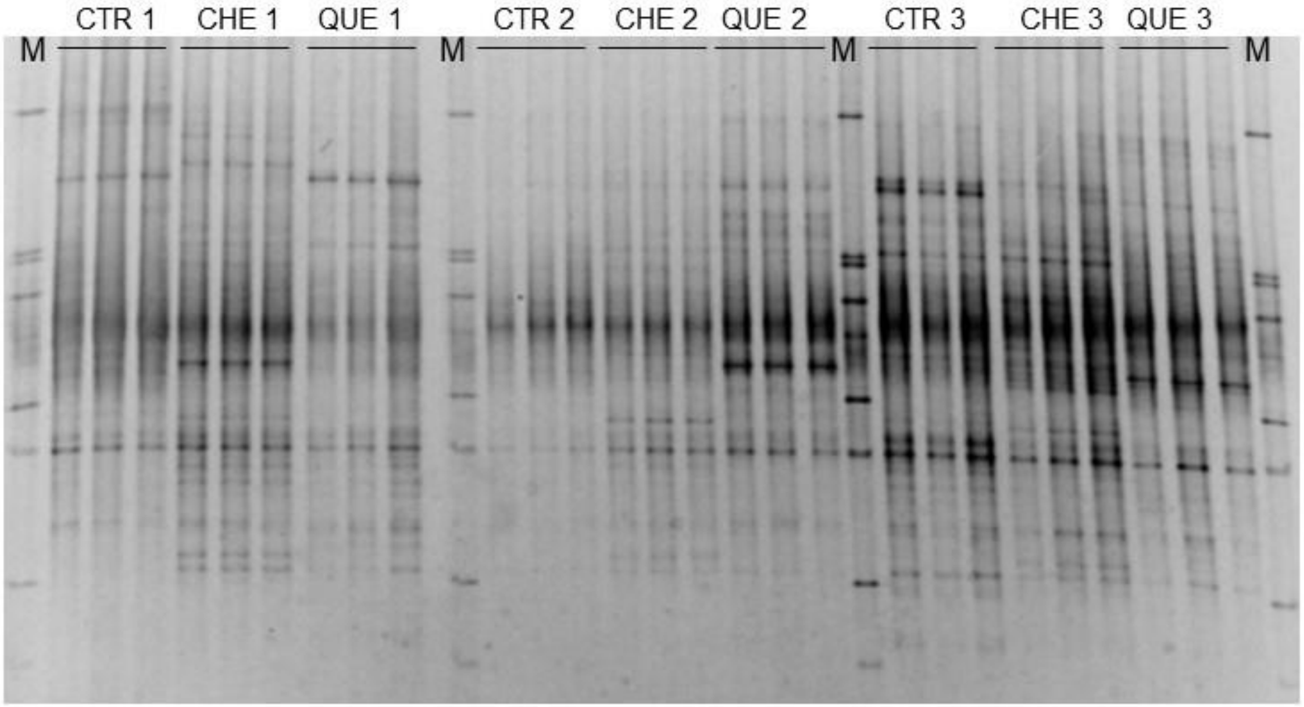

Supplementary material 2. DGGE profiles of 16S rDNA PCR products obtained from DNA extracted from rumen liquor using primer for the *Butyrivibrio* group (F968GC-B fib). CTR, control diet (84 g kg^{-1} DM of soybean oil); CHT, chestnut tannins diet (84 g kg^{-1} DM of soybean oil plus 52.8 g kg^{-1} DM of a chestnut tannin extract); QUE, quebracho tannins diet (84 g kg^{-1} DM of soybean oil plus 52.8 g kg^{-1} DM of a quebracho tannin extract); M, marker used for normalization of bands.
